# Communication Skills in Medical Education and Practice: A Bibliometric Analysis of the Current Research Landscape

**DOI:** 10.7759/cureus.104018

**Published:** 2026-02-21

**Authors:** Amit K Pal, Dyutimoy Datta, Mutyalapati Venkata Ramulu, Vaishnavi Agnihotri

**Affiliations:** 1 Department of Anatomy, All India Institute of Medical Sciences, Kalyani, Kalyani, IND; 2 Department of Anatomy, Mahabodhi Medical College and Hospital, Gaya Ji, IND

**Keywords:** bibliometric analysis, communication skills, doctor-patient communication, medical education, patient education

## Abstract

Communication skills are widely recognized as a core competency in medical education and clinical care, as they impact professional effectiveness, patient experience, and health outcomes. Over the past two decades, research in this area has expanded rapidly, creating the need for a comprehensive mapping of the field. To provide a global bibliometric analysis of research on communication skills in medical education and clinical practice published between 2000 and 2025, a systematic bibliometric search of PubMed (MEDLINE) and the Web of Science Core Collection was conducted for the period January 2000 to mid-2025. After deduplication and screening, 14,829 publications were included. Bibliometric indicators were analyzed using the Bibliometrix package in R and VOSviewer to evaluate publication trends, authorship patterns, journal impact, keyword co-occurrence, thematic evolution, and international collaboration networks. Annual publication output increased approximately 15-fold, from 86 publications in 2000 to 1,344 in 2025. Research was concentrated in a limited number of high-impact journals, led by Patient Education and Counseling, BMC Medical Education, Medical Teacher, and Medical Education. The United States, the United Kingdom, Germany, China, Canada, and Australia emerged as dominant contributors, with increasing international collaboration over time. Keyword and thematic analyses identified four major clusters: clinician-patient communication and outcomes, medical education and assessment, communication in sensitive and high-stakes clinical contexts, and emerging digital and technology-mediated communication modalities. Research on communication skills has evolved into a mature, interdisciplinary, and globally distributed field. The findings demonstrate strong curricular integration, increasing methodological sophistication, and growing attention to digital transformation while also highlighting persistent geographic and contextual gaps that warrant further research and educational innovation.

## Introduction and background

Effective clinician-patient communication is widely recognized as a cornerstone of high-quality healthcare and professional medical practice [1]. Communication skills underpin essential clinical activities, including history-taking, explanation of diagnoses and treatment options, risk disclosure, informed consent, shared decision-making, bereavement support, and follow-up care [2,3]. Beyond interpersonal exchange, communication has demonstrable effects on diagnostic accuracy, treatment adherence, patient satisfaction, psychological well-being, and patient safety [4-7]. Conversely, ineffective communication is associated with preventable medical errors, patient dissatisfaction, and increased malpractice risk, underscoring its clinical and ethical significance [8].

Historically, communication was regarded as an implicit attribute acquired through apprenticeship and observation of senior clinicians. This informal model offered limited opportunities for structured instruction, deliberate practice, or formal assessment [9]. Over time, empirical evidence demonstrating that communication is teachable, measurable, and directly linked to patient outcomes prompted a paradigm shift toward structured educational approaches [10,11]. The introduction of the Objective Structured Clinical Examination (OSCE) provided a methodological foundation for assessing communication as a clinical competency in standardized, reproducible settings [12,13].

The formalization of communication training was further advanced by consensus-based educational frameworks. The Calgary-Cambridge Guides offered a structured model of the clinical consultation, integrating content and process across all phases of the encounter [14,15]. Similarly, the Kalamazoo Consensus Statement defined essential elements of clinician-patient communication and provided a shared curricular language for teaching and assessment [16]. These developments supported the transition from episodic workshops to longitudinal, competency-based communication curricula embedded throughout undergraduate and postgraduate medical education.

Regulatory and accreditation bodies subsequently reinforced the centrality of communication skills. Interpersonal and communication skills were designated as a core competency by the Accreditation Council for Graduate Medical Education in the United States, while the General Medical Council in the United Kingdom mandated structured communication training within the medical curricula [17-19]. In India, the National Medical Commission introduced the longitudinal Attitude, Ethics, and Communication (AETCOM) modules as part of competency-based medical education, emphasizing the formation of professional identity alongside technical competence [20-22].

Despite these advances, notable disparities persist in the global research landscape. Bibliometric studies have shown that communication skills research is disproportionately produced by institutions in North America and Western Europe, with relatively limited representation from low- and middle-income countries [23,24]. This imbalance has implications for the generalizability of dominant communication models, which are often rooted in Western cultural assumptions about autonomy, authority, and decision-making [25]. As communication is inherently context dependent, there is a growing need to examine how established frameworks translate across diverse healthcare systems, cultures, and resource settings.

Given the substantial growth, thematic diversification, and global relevance of scholarship on communication skills, a comprehensive bibliometric analysis is warranted. Bibliometric methods enable the systematic mapping of publication trends, influential contributors, collaborative networks, and evolving research themes, thereby offering insights beyond those from narrative reviews alone. This study, therefore, aims to map the global research landscape on communication skills in medical education and clinical practice over the past 25 years. The objectives of this analysis are to quantify publication growth and citation patterns from 2000 to 2025, identify leading journals, authors, institutions, and countries, map thematic evolution and keyword-based research clusters, and examine international collaboration patterns and geographic distribution.

Methods

Data Sources and Search Strategy

A comprehensive bibliometric search was conducted using two major bibliographic databases: PubMed (MEDLINE) and the Web of Science Core Collection. These databases were selected to ensure broad coverage of peer-reviewed biomedical, clinical, and medical education literature while also enabling robust citation and network analyses. The search period spanned from January 2000 to mid-2025, reflecting the period during which communication skills became formally embedded within competency-based medical education and clinical training frameworks.

The search strategy (Table 1) was designed to maximize sensitivity while maintaining relevance to communication skills in medical education and clinical practice. Controlled vocabulary terms (Medical Subject Headings (MeSH)) were used in PubMed, and free-text keyword searches were applied in Web of Science. Search terms covered three conceptual domains: (i) medical and clinical contexts (e.g., medical education, physician, clinician, medical student, resident), (ii) communication constructs (e.g., communication skills, doctor-patient communication, interpersonal communication, empathy, counselling, breaking bad news, shared decision-making), and (iii) educational and evaluative dimensions (e.g., teaching, learning, assessment, training, competence, outcomes, implementation). Database-specific syntax and field tags were applied, and the final search strings were iteratively refined to balance completeness with specificity and to minimize the retrieval of non-clinical or non-medical communication literature. 

**Table 1:** Literature search strategy

Databases searched	PubMed (MEDLINE); Web of Science Core Collection
Time frame	January 2000 to mid-2025
Search terms	• Medical education–related terms (medical education, medical teaching, medical learning, medical training, medical curriculum)
	• Medical trainees and professionals (medical students, undergraduate and postgraduate medical students, medical graduates, interns, resident doctors, junior doctors, house officers, medical trainees, medical practitioners, physicians, clinicians, doctors, medical professionals, healthcare providers, health professionals, medical faculty, medical teachers)
	• Communication skills–related terms (communication skills, doctor–patient communication, physician–patient communication, clinician–patient communication, consultation skills, interpersonal communication, clinical communication, therapeutic communication, prescription communication, communication competence, communication effectiveness, communication training, communication teaching, communication learning, communication curriculum)
	• Ethics- and empathy-focused communication (AETCOM, attitude, ethics and communication, empathic communication, empathy, patient-centred communication, professional communication, medical communication)
	• Interpersonal and soft skills (interpersonal skills, soft skills, counselling skills, verbal communication, non-verbal communication, rapport building)
	• implementation and outcome (perception, attitude, awareness, belief, experience, feedback, reflection, teaching, learning, assessment, evaluation, training, competence, skill development, impact, effectiveness, outcomes, challenges, barriers, facilitators, implementation, principles, practices)
Boolean operators	AND, OR (used to combine and conceptualize search concepts); NOT (used to exclude non-relevant disciplines)
Filters applied	English language; human studies
Screening criteria	Title and abstract screening followed by eligibility assessment based on predefined inclusion and exclusion criteria
Supplementary search	Manual screening of reference lists from the included articles
Exclusion criteria	Studies focused primarily on dentistry, dental students, nursing, nurses, paramedical disciplines, pharmacy, veterinary medicine, physiotherapy, allied health professions, or non-medical communication contexts
Final dataset	14,829 publications included for bibliometric analysis

All records retrieved from the Web of Science were exported as BibTeX files using the "Full Record and Cited References" option to preserve bibliographic and citation metadata required for co-citation, bibliographic coupling, and network analyses. PubMed records were exported as delimited text files, capturing titles, abstracts, indexing terms, and bibliographic metadata. The exported datasets were imported into Biblioshiny (Bibliometrix package, R), where records from both databases were merged and standardized.

Following database integration, duplicate records were identified and removed using a combination of automated matching (based on title, authors, and publication year) and manual verification. After deduplication, the cleaned dataset was exported for screening and further refinement. Core bibliometric fields retained for analysis included authorship information, publication year, journal/source title, abstracts, author keywords and MeSH terms (where available), author affiliations, and cited references.

Screening and Eligibility Criteria

To ensure that the analytic dataset reflected literature explicitly focused on communication skills in medical education and clinical practice, a two-stage screening process was undertaken. Two researchers independently screened titles and abstracts using predefined inclusion and exclusion criteria. Since the eligibility criteria were primarily based on relevance to communication skills within medical education and clinical practice, the screening process was designed to ensure topical alignment rather than the interpretive assessment of study quality or outcome direction, as would typically be undertaken in an interventional systematic review. Given the bibliometric nature of the study, a formal Cohen's kappa statistic was not calculated at the time of screening. Disagreements between reviewers were infrequent and resolved through structured consensus discussion.

Studies were included if they met at least one of the following criteria: (i) addressed principles, frameworks, or practices of communication skills in medical education or clinical medicine; (ii) examined the teaching, learning, or assessment of communication skills among medical students, residents, or practicing physicians; (iii) evaluated outcomes or effectiveness of communication skills training or interventions; or (iv) explored barriers, facilitators, or implementation strategies related to communication in medical settings.

Studies were excluded if they were not published in English or if communication was only peripherally addressed, such as communication in non-medical professions, non-clinical settings, or allied health disciplines without direct relevance to physician-patient interaction.

**Figure 1:**
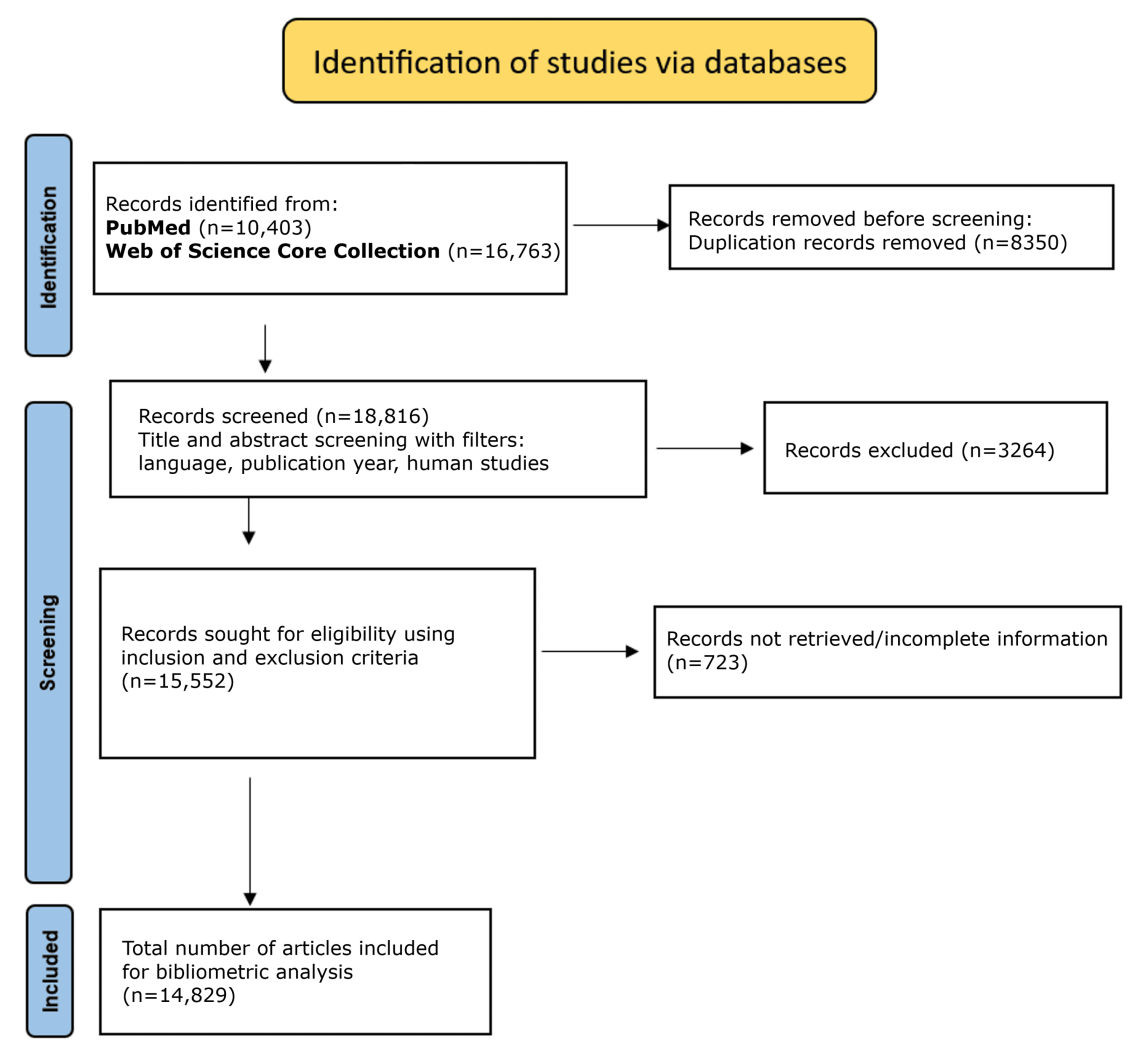
PRISMA flowchart showing the selection process of articles for bibliometric analysis PRISMA: Preferred Reporting Items for Systematic Reviews and Meta-Analyses

Following screening, a final analytic dataset of 14,829 publications was obtained and used for bibliometric analysis. The study selection process was documented using a Preferred Reporting Items for Systematic Reviews and Meta-Analyses (PRISMA)-style flow diagram (Figure 1).

Bibliometric Analysis and Visualization

Descriptive bibliometric indicators were generated using the Bibliometrix package (version 3.2) in R, including annual publication trends, most productive authors, institutions, countries, and journals, as well as citation-based impact measures such as h-index and g-index at the source level. Science mapping techniques were applied to examine intellectual and social structures within the field.

Keyword and thematic mapping and collaboration network analyses were conducted using Bibliometrix, while source and reference co-citation analyses were carried out using VOSviewer (version 1.6.18). Visualizations were generated using standard thresholds for minimum occurrence and link strength to enhance interpretability while preserving analytical robustness.

Bibliometric Analysis Tools

We utilized the Bibliometrix R package (version 3.2) for comprehensive bibliometric analysis. Bibliometrix facilitated performance analysis (e.g., publication counts by year, most prolific authors, institutions, journals) and science mapping. We also used VOSviewer (version 1.6.18) for network analyses and visualization of co-authorship and keyword co-occurrence. For network visualizations, threshold parameters were defined in advance to ensure clarity while maintaining analytical integrity. A minimum occurrence threshold of 10 was applied for keyword co-occurrence analyses, and a citation-based cut-off threshold of 5 was used for source and reference co-citation mapping to improve the readability of the networks. Unless otherwise specified, default normalization and clustering settings in VOSviewer (version 1.6.18) were applied. The choice of these tools was informed by their common use in bibliometric studies.

## Review

Results

Publication Trends (2000-2025)

**Figure 2:**
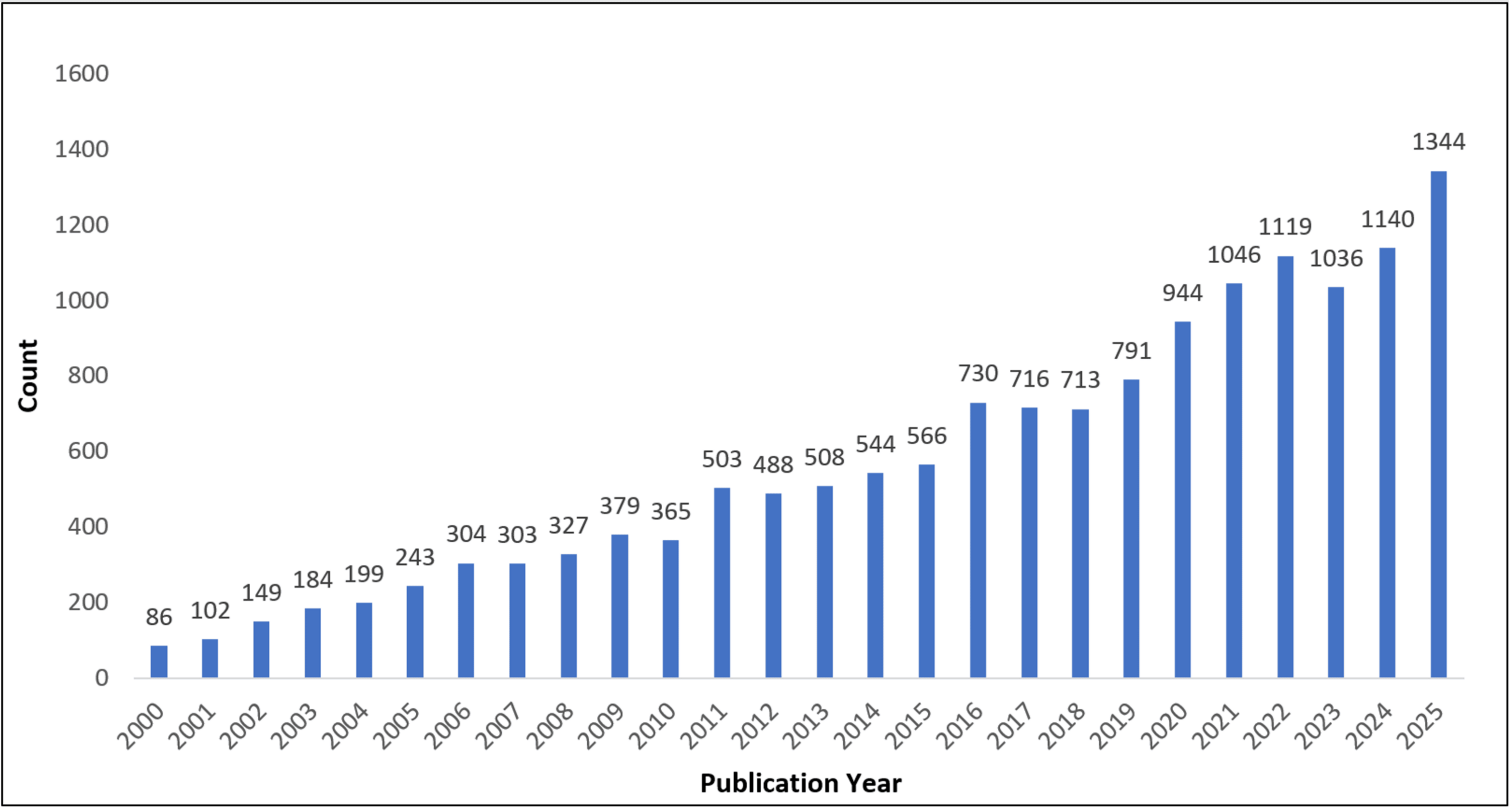
Publication trends (years 2000-2025)

Following deduplication and screening, a total of 14,829 publications from 2000 to mid-2025 were included in the final bibliometric dataset. As observed in Figure 2, the analysis of annual publication output demonstrated a pronounced and sustained growth in research on communication skills in medical education and clinical practice over the 25-year period. The number of publications increased from 86 in 2000 to 1,344 in 2025, representing an approximately 15-fold rise. The field has experienced a compound annual growth rate (CAGR) of about 11.6%, which reflects consistent double-digit growth over the past 25 years. Growth was relatively gradual during the early 2000s, followed by a marked acceleration after 2010. A further surge was observed between 2016 and 2021, coinciding with the global expansion of competency-based medical education, increasing emphasis on assessment, and heightened interest in telemedicine and remote communication during the COVID-19 pandemic.

Journal Distribution and Source Impact

**Figure 3:**
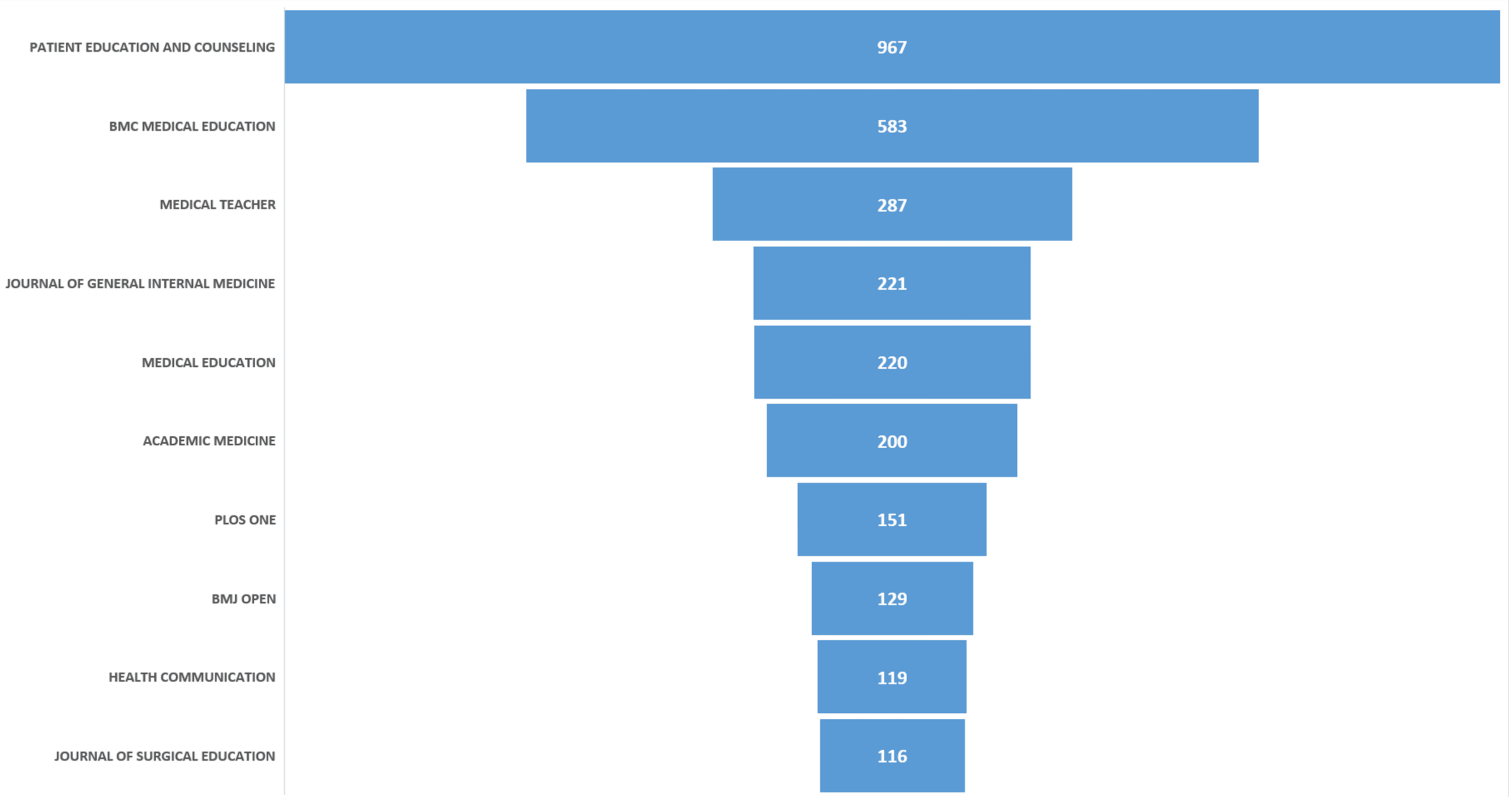
Top ranked journals

As depicted in Figure 3, the 14,829 publications were distributed across more than 1,500 peer-reviewed journals, reflecting the interdisciplinary nature of communication skills research. A small group of journals accounted for a substantial proportion of the output. Patient Education and Counseling emerged as the most prolific source, contributing 967 publications (approximately 6.5% of the dataset), followed by BMC Medical Education (583 publications), Medical Teacher (287), Journal of General Internal Medicine (221), Medical Education (220), and Academic Medicine (200). This distribution indicates that communication skills research is anchored in both dedicated health communication journals and leading medical education and clinical journals.

Source-Level Impact Analysis

**Figure 4:**
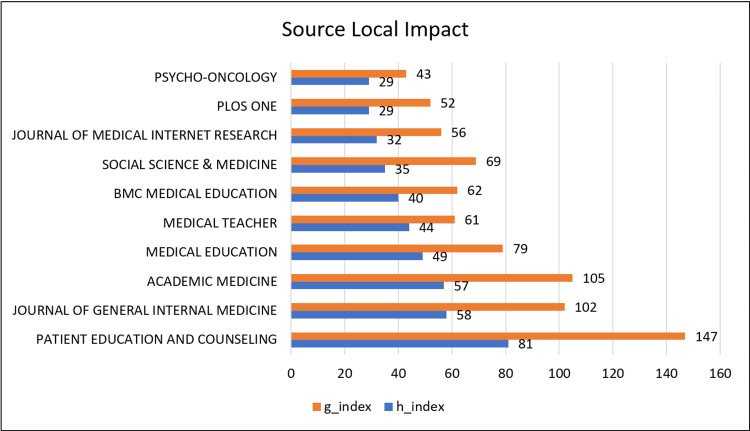
Journal impact

Figure 4 further demonstrates the dominance of a limited number of journals. Patient Education and Counseling showed the highest local h-index and g-index values, indicating both high productivity and sustained citation impact within the field. Other journals with strong impact metrics included Journal of General Internal Medicine, Academic Medicine, and Medical Education, underscoring their central role in disseminating influential scholarship on communication skills.

Document Types

**Figure 5:**
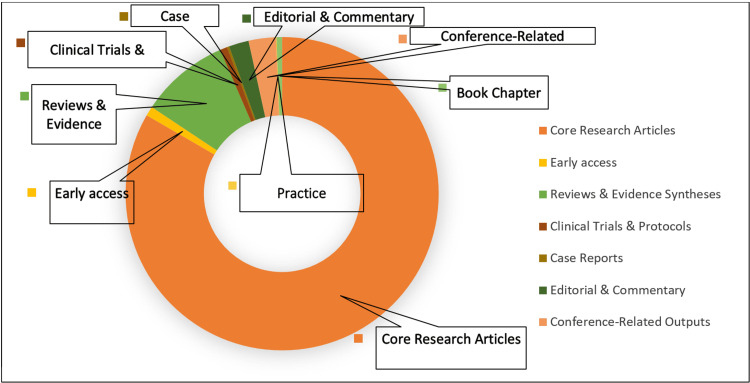
Distribution of types of published documents

As seen in Figure 5, original research articles constituted the majority of publications in the dataset, indicating that the field is primarily driven by empirical investigation. Reviews and evidence syntheses formed a smaller but substantial proportion, reflecting efforts to consolidate emerging knowledge and inform educational practice. Editorials, commentaries, and conference-related publications were present in limited numbers, while clinical trials and protocol-based studies remained relatively underrepresented, suggesting opportunities for future methodological diversification.

Authorship Patterns and Prolific Contributors

**Figure 6:**
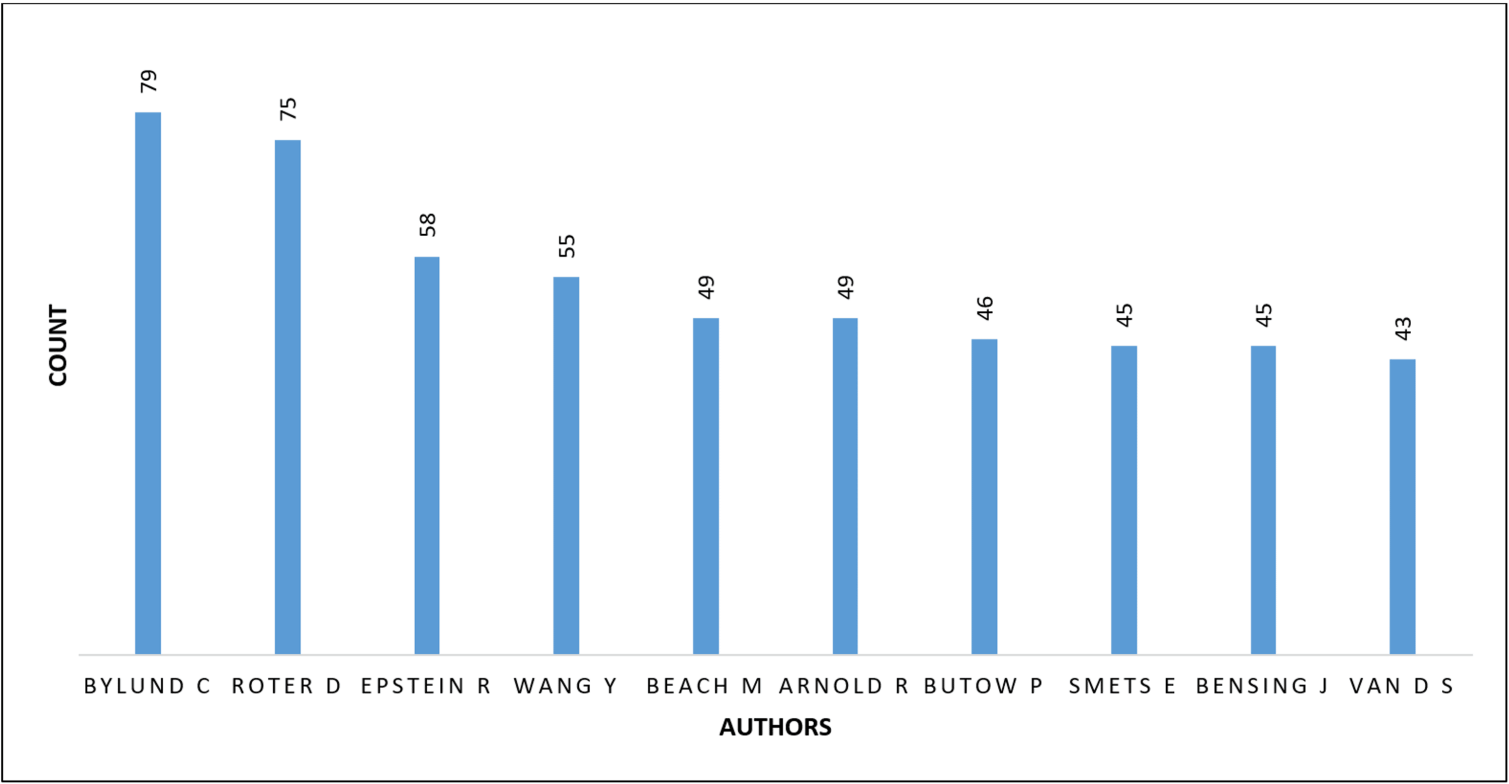
Top contributing authors Bylund C: Carma L. Bylund; Roter D: Debra L. Roter; Epstein R: Ronald M. Epstein; Wang Y: Yan Wang; Beach M: Mary Catherine Beach; Arnold R: Robert Arnold; Butow P: Phyllis N. Butow; Smets E: Ellen M.A. Smets; Bensing J: Jozien M. Bensing; van D S: A.M. (Sandra) van Dulmen

A total of 36,123 unique authors contributed to the included publications, highlighting the broad scholarly engagement with communication skills research. The median number of authors per article increased over time, from approximately two authors per paper in the early 2000s to four or more authors in recent years, indicating a shift toward more collaborative research practices as shown in Figure 6.

Analysis of author productivity identified a small group of highly prolific contributors. The most productive authors over the study period included Carma L. Bylund, Debra L. Roter, Ronald M. Epstein, Yinghui Wang, and Mary C. Beach, all of whom have made sustained contributions to the development of communication skills research in medical education and clinical contexts. Citation analysis further demonstrated that highly cited publications were predominantly conceptual or framework-defining works, particularly those related to consultation models, curriculum integration, and assessment strategies.

Highly Cited Publications and Intellectual Foundations

**Figure 7:**
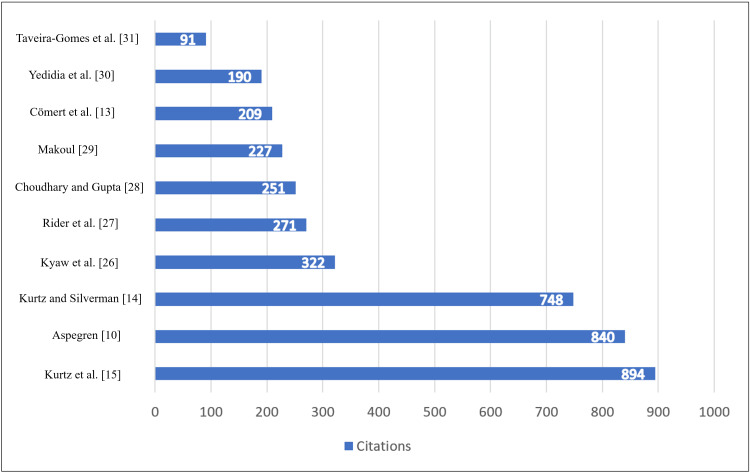
Top-cited articles with the respective authors' names

The dataset's citation profile in Figure 7 and Table 2 revealed a concentrated intellectual core. Seminal works by Kurtz and Silverman on the Calgary-Cambridge framework and by Aspegren on teaching and learning communication skills were among the most highly cited publications. Influential studies from the early 2000s established communication skills as teachable and assessable competencies, while more recent highly cited works focused on assessment rigor, digital education, and technology-enhanced learning. This pattern reflects a gradual shift from foundational theory-building toward applied and evaluative research.

**Table 2:** Most cited publications

Author(s)	Year	Citations	Article title
Kurtz et al. [15]	2003	894	Marrying content and process in clinical method teaching: enhancing the Calgary-Cambridge guides
Aspegren [10]	1999	840	BEME Guide No. 2: teaching and learning communication skills in medicine-a review with quality grading of articles
Kurtz and Silverman [14]	1996	748	The Calgary-Cambridge referenced observation guides: an aid to defining the curriculum and organizing the teaching in communication training programmes
Kyaw et al. [26]	2019	322	Effectiveness of digital education on communication skills among medical students: systematic review and meta-analysis by the Digital Health Education Collaboration
Rider et al. [27]	2006	271	A model for communication skills assessment across the undergraduate curriculum
Choudhary and Gupta [28]	2015	251	Teaching communications skills to medical students: introducing the fine art of medical practice
Makoul [29]	2003	227	Communication skills education in medical school and beyond
Cömert et al. [13]	2016	209	Assessing communication skills of medical students in objective structured clinical examinations (OSCE) - a systematic review of rating scales
Yedidia et al. [30]	2003	190	Effect of communications training on medical student performance
Taveira-Gomes et al. [31]	2016	91	Communication skills in medical students - an exploratory study before and after clerkships

Institutional Affiliations and Geographic Distribution

**Figure 8:**
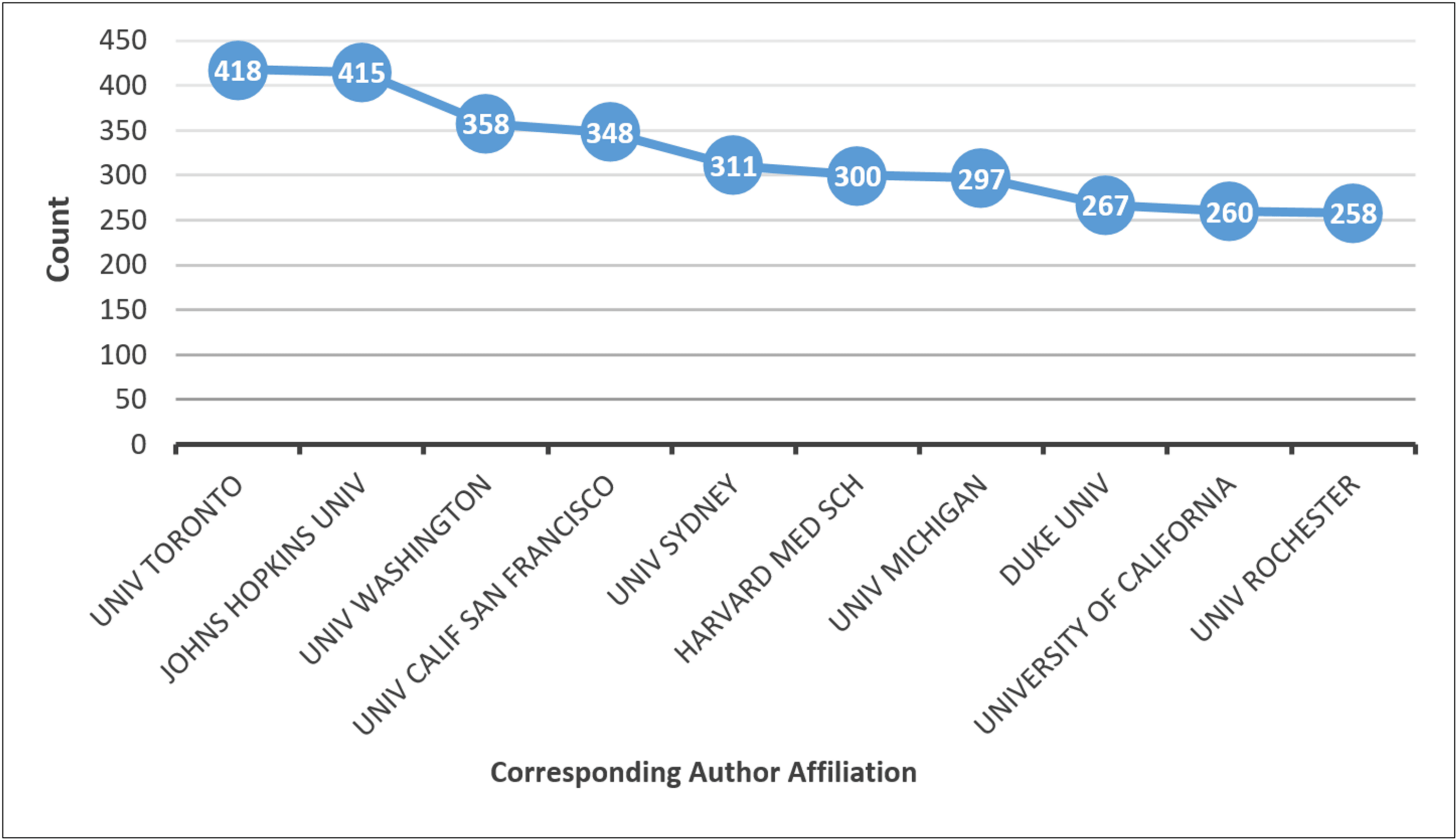
Corresponding authors' affiliations Univ Toronto: University of Toronto; Johns Hopkins Univ: Johns Hopkins University; Univ Washington: University of Washington; Univ Calif San Francisco: University of California, San Francisco; Univ Sydney: University of Sydney; Harvard Med Sch: Harvard Medical School; Univ Michigan: University of Michigan; Duke Univ: Duke University; University of California: University of California, San Diego; Univ Rochester: University of Rochester

Analysis of corresponding author affiliations in Figure 8 showed that a limited number of institutions contributed disproportionately to the literature. Universities in North America and Europe dominated the list of top-affiliated institutions, with the University of Toronto and Johns Hopkins University among the most productive. Other leading institutions included the University of Washington, the University of California (San Francisco), and the University of Sydney.

Geographic Distribution Analysis

**Figure 9:**
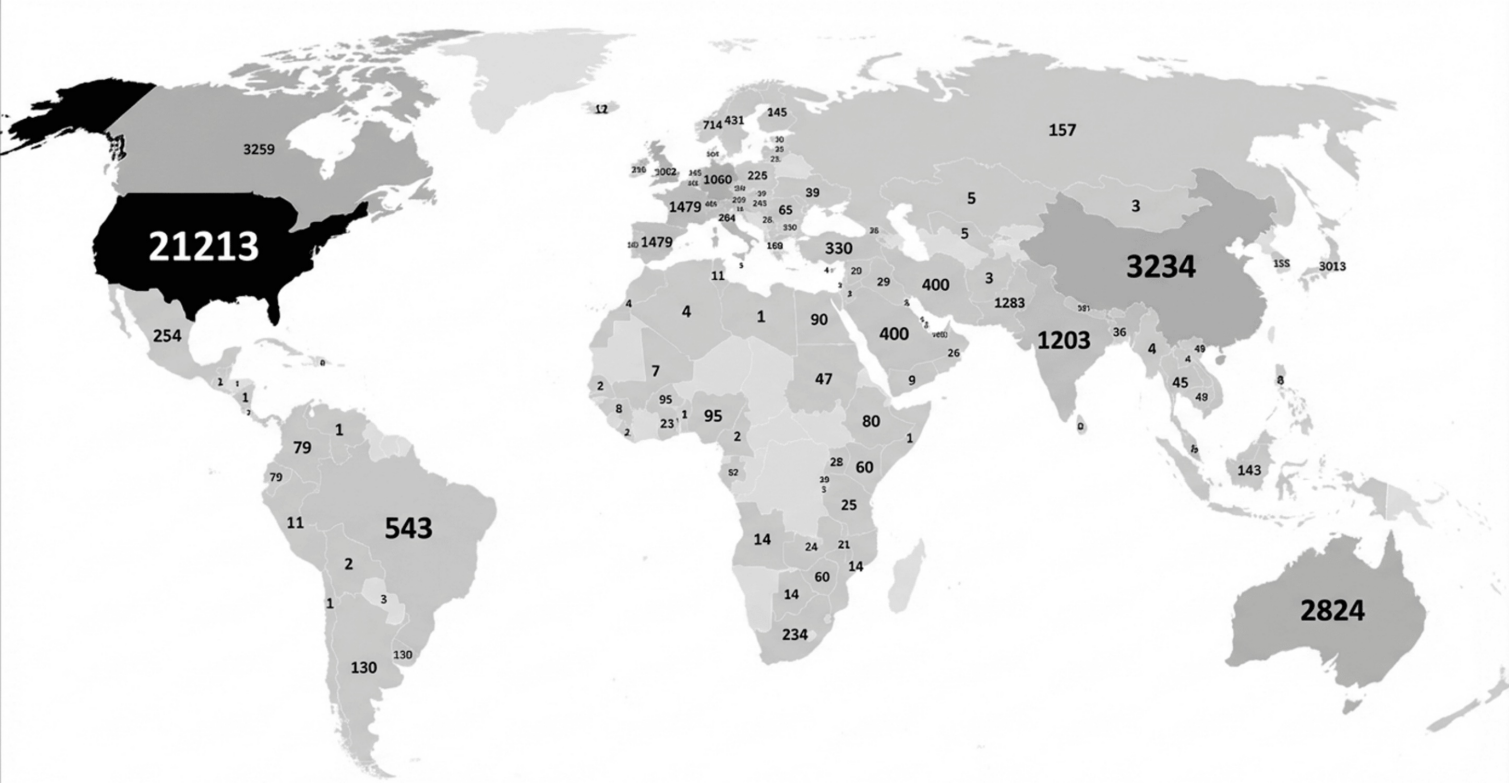
Geographic distribution of research The intensity of colors ranging from more dark (black) to less dark (white) explains the country-wise contributions of the number of articles. More dark (black): more contribution; less dark (white): less contribution

**Figure 10:**
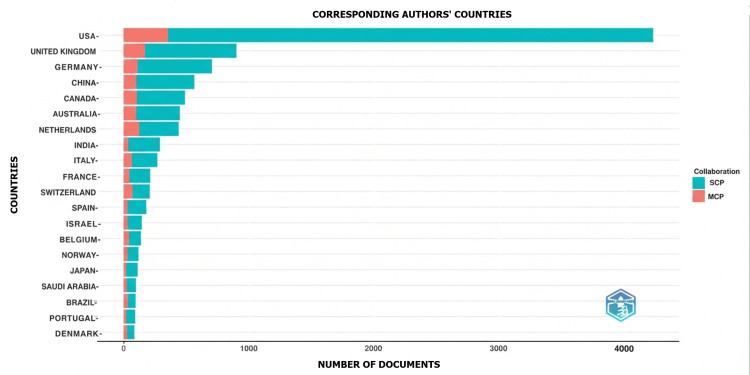
Proportion of collaboration from the top contributing countries SCP: single-country publications; MCP: multiple-country publications

Geographic analysis demonstrated that communication skills research has a global footprint, with contributions from more than 100 countries. The United States accounted for the largest share of publications, followed by the United Kingdom, Germany, China, Canada, and Australia. While single-country publications predominated, multiple-country publications increased over time, indicating growing international collaboration, particularly among high-income countries as shown in Figures 9-10.

Keyword Co-occurrence and Thematic Structure

**Figure 11:**
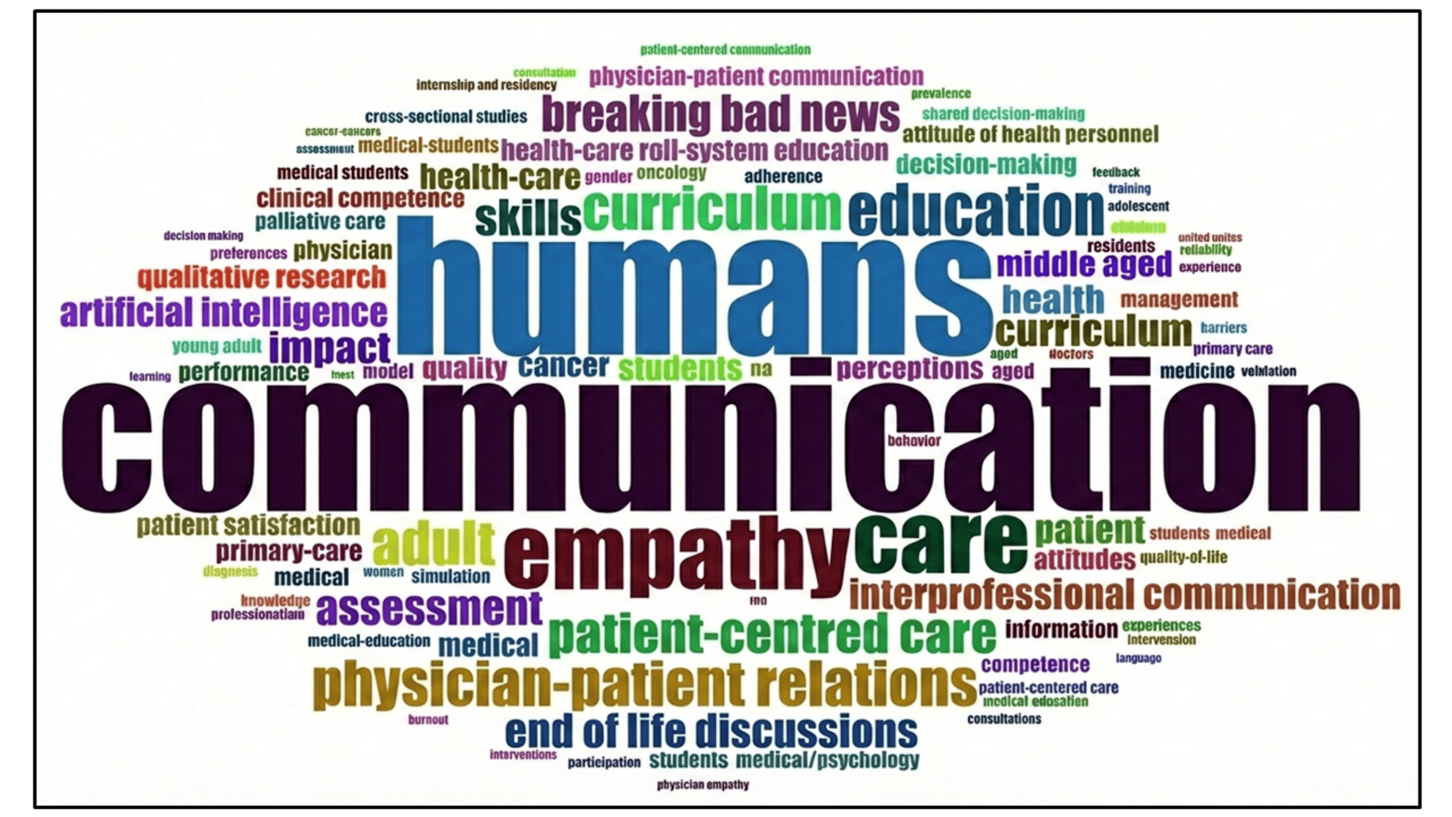
Keyword analyses

**Figure 12:**
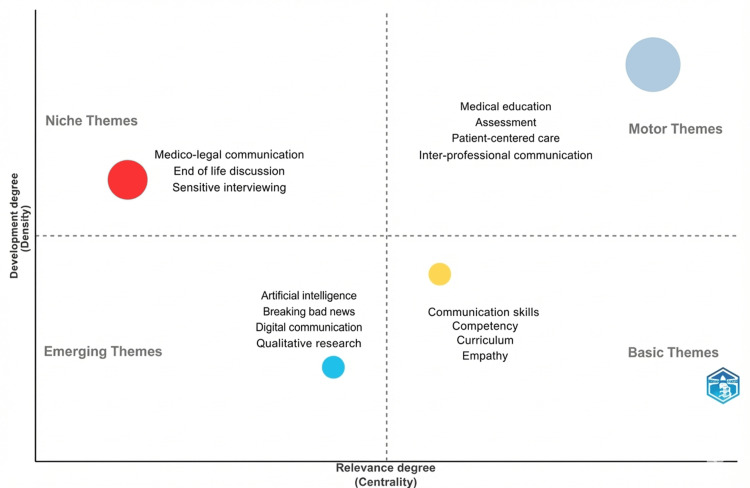
Thematic analyses

Keyword co-occurrence analysis in Figure 11 revealed that core terms such as "communication", "medical education", "empathy", "patient-centered care", and "assessment" formed the backbone of the research landscape. Thematic mapping in Figure 12 identified four major thematic clusters. Motor themes, characterized by high centrality and density, included medical education, assessment, and patient-centered communication, reflecting well-developed and influential areas of research. Basic themes such as communication skills, competency, curriculum, and empathy were widely connected but conceptually broad. Emerging themes encompassed digital communication, artificial intelligence, qualitative research, and breaking bad news, indicating areas of growing scholarly interest. Niche themes, including medico-legal communication and end-of-life discussions, represented specialized but relatively isolated research domains.

Source and Reference Co-citation 

**Figure 13:**
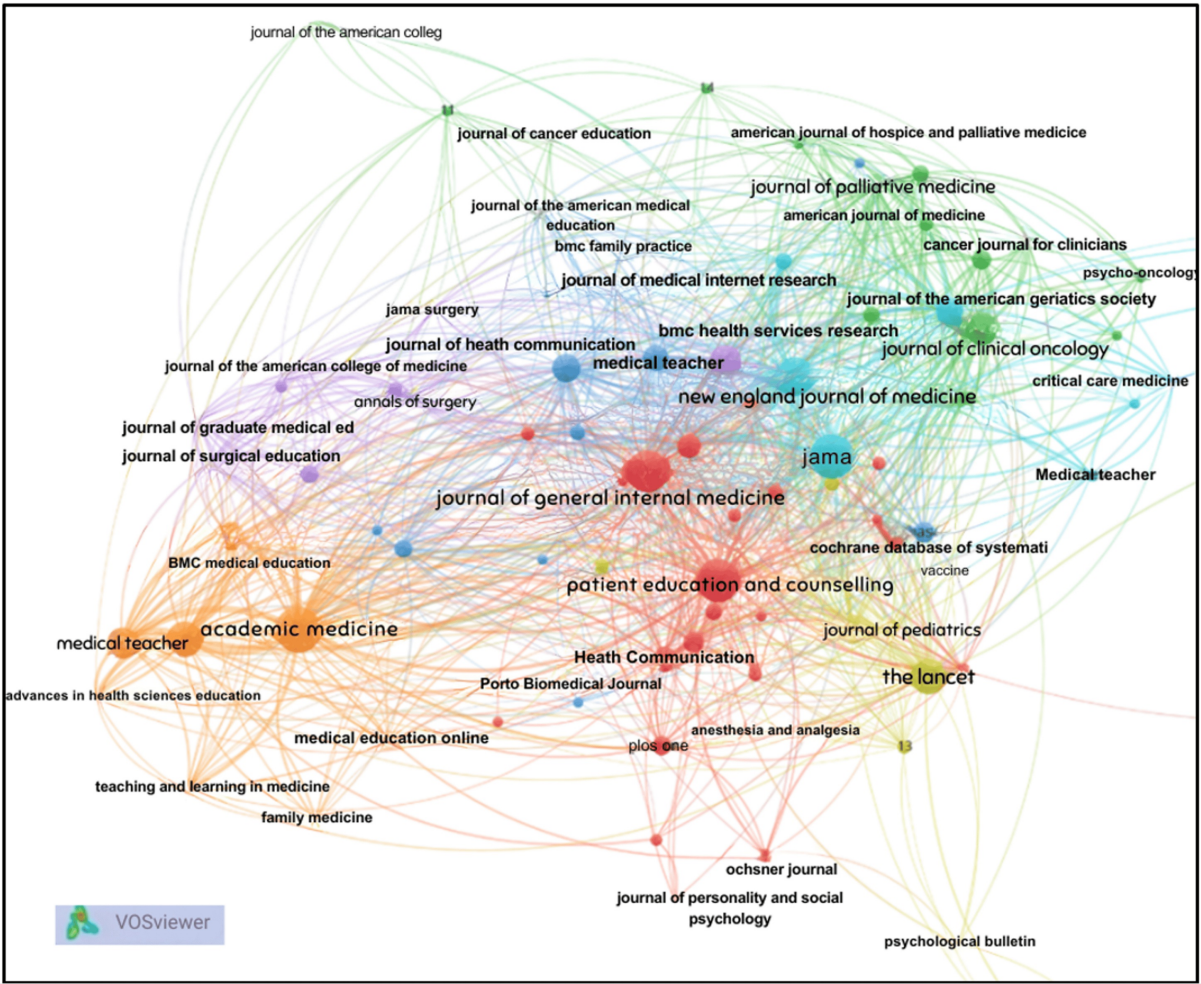
Network map of the source co-citation of research The figure was created using VOSviewer.

**Figure 14:**
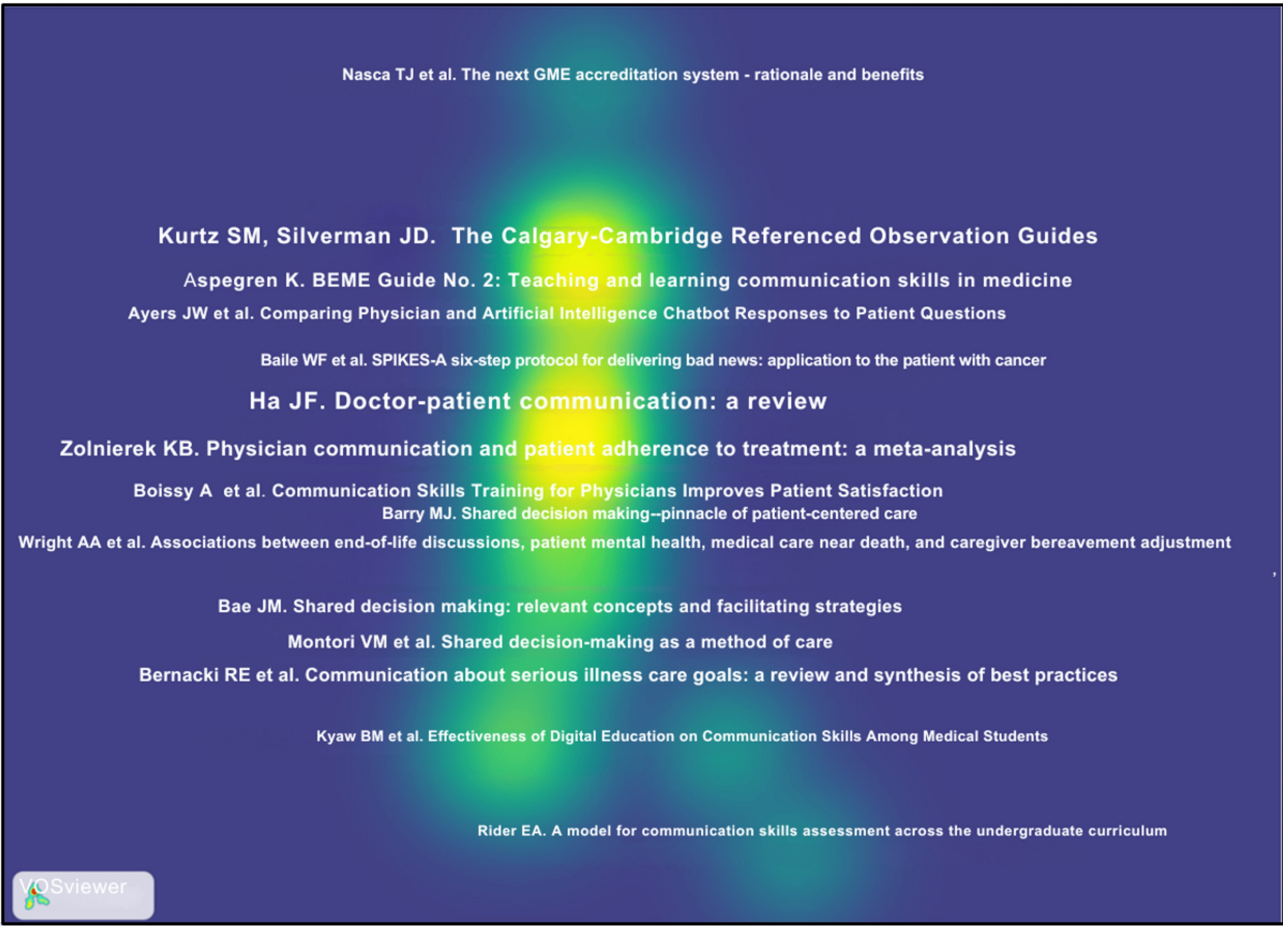
Density visualization of the reference co-citation The figure was created using VOSviewer.

Source co-citation analysis in Figure 13 identified a concentrated core of highly influential journals, including Patient Education and Counseling, Journal of General Internal Medicine, JAMA, The Lancet, New England Journal of Medicine, Academic Medicine, and Medical Education. The density visualization heat map in Figure 14 highlights a solid intellectual core in research on communication skills within medical education and practice. The brightest areas center around key references, such as the Calgary-Cambridge Referenced Observation Guides and Ha and Longnecker's review on doctor-patient communication, indicating they serve as essential anchors in the field. Surrounding this core are moderately dense zones related to shared decision-making, end-of-life communication, and assessment frameworks, suggesting a strong link to foundational communication theory. In contrast, lower-density areas, like digital communication and artificial intelligence, appear at the periphery, representing newer topics beginning to connect with established literature.

Discussion

This bibliometric analysis maps 25 years (2000-2025) of scholarship on communication skills in medical education and clinical practice and demonstrates the evolution of the field from advocacy-oriented discourse to a consolidated, outcomes-focused research domain. The marked growth in publication volume, the concentration of output within a core group of high-impact journals, and the emergence of stable thematic clusters together indicate that communication skills are now firmly established as a core clinical competency rather than a peripheral or informal attribute of medical practice [3,24,32,33].

The approximately 15-fold increase in annual publications observed between 2000 and 2025 reflects both empirical and structural drivers. Accumulating evidence linking clinician-patient communication with treatment adherence, patient satisfaction, safety, and medico-legal outcomes provided a strong rationale for sustained scholarly attention [4,5,8,34]. In parallel, the formal incorporation of communication skills into accreditation standards and competency-based curricula created an enabling environment for educational research. As a result, the literature has progressively shifted from establishing the importance and teachability of communication toward examining implementation strategies, assessment practices, and educational effectiveness [9,11,35].

Journal and citation analyses further illustrate the field's consolidation around a small number of influential publication venues. The prominence of Patient Education and Counseling, Medical Education, Academic Medicine, and BMC Medical Education underscores the interdisciplinary positioning of communication skills research at the intersection of clinical practice, health services research, and medical education [24,32]. The sustained citation impact of foundational frameworks, particularly the Calgary-Cambridge Guides and the Kalamazoo Consensus Statement, indicates continued reliance on shared conceptual models that have shaped curriculum design, assessment, and research agendas over multiple decades [15,16].

Authorship and collaboration patterns reveal a dense core of recurring contributors and institutions, predominantly based in North America and Western Europe, alongside expanding but less integrated participation from other regions. While such a structure facilitates methodological continuity and cumulative knowledge building, it also highlights enduring inequities in global knowledge production. Given that communication practices are deeply embedded in cultural norms, healthcare structures, and social expectations, the relative underrepresentation of low- and middle-income countries raises important questions about the contextual validity and transferability of dominant communication models [23-25,36,37]. The findings therefore reinforce calls for more locally led and context-sensitive research to complement existing frameworks.

Thematic evolution analyses demonstrate a clear progression from early emphasis on discrete communication techniques toward broader relational and patient-centred competencies. Initial research focused on consultation structure, information exchange, and observable micro-skills, whereas later work increasingly addressed empathy, shared decision-making, and the relational dimensions of care [3,11,36,38]. This shift mirrors wider changes in clinical practice, including movement away from paternalistic models toward partnership-based care, and reflects growing recognition that effective communication involves professional identity, values, and judgment rather than behavioral checklists alone [14,38].

Assessment-focused scholarship represents a further marker of disciplinary maturation. The widespread adoption of OSCE-based methods normalized communication as a measurable and accountable competency, generating a substantial body of research on rating scales, reliability, and validity [12,13,39]. At the same time, the literature increasingly acknowledges limitations of simulation-based assessment, particularly regarding the durability of learning and transfer to real clinical environments. These findings have prompted growing interest in workplace-based assessment, longitudinal feedback, and coaching models that align assessment more closely with authentic clinical practice [13,16,40].

Curricular trends identified through bibliometric patterns align closely with policy and regulatory developments across regions. The expansion of structured training for high-stakes conversations, especially in oncology and palliative care, reflects the translation of clinical need into targeted educational interventions [41,42]. In India, the introduction of the AETCOM modules represents a systematic attempt to integrate communication longitudinally within competency-based medical education. While early evaluations indicate favorable learner perceptions, the limited number of outcome-focused studies highlights the need for more robust evidence on educational impact and contextual adaptation [20,21].

More recent thematic clusters capture the influence of digital transformation on communication education and practice. The COVID-19 pandemic accelerated the adoption of telemedicine and remote teaching, drawing attention to communication competencies specific to virtual encounters, including managing reduced non-verbal cues and maintaining patient-centredness in digitally mediated settings [43,44]. Emerging research on artificial intelligence and large language models suggests potential roles in simulation, rehearsal, and feedback while also raising ethical and governance concerns related to accuracy, trust, and professional responsibility [45,46]. Although these topics currently occupy peripheral positions within the broader literature, their rapid growth indicates an important trajectory for future research.

Overall, the integrated findings suggest that communication skills research has achieved conceptual stability while continuing to expand in scope and methodological diversity. The overall citation and thematic patterns suggest that much of the highly influential literature focuses on the validation of teaching models and the demonstration of favorable training outcomes. Reporting of null or negative findings appears less prominent within the most cited work. Future advancement of the field will depend on addressing persistent geographic imbalances, strengthening evidence on long-term transfer of training effects to clinical practice, and systematically evaluating communication competencies within increasingly digital healthcare environments. Such efforts are essential to ensure that communication training remains educationally robust, culturally responsive, and aligned with evolving models of care delivery.

Limitations

This bibliometric analysis has several limitations. First, the dataset was restricted to publications indexed in PubMed and the Web of Science Core Collection and to articles published in English, which may have excluded relevant studies indexed elsewhere or published in other languages. Second, bibliometric indicators primarily reflect publication and citation patterns and cannot directly assess the methodological quality or educational effectiveness of individual studies. Third, citation counts may underestimate the influence of more recent publications due to citation lags. These limitations should be considered when interpreting the findings.

Implications for future research

Several important priorities emerge from this bibliometric analysis. First, equity in research output in the concerned matter should be recognized as an essential requirement rather than an optional consideration. Communication models developed primarily in Western settings need to be tested across diverse cultural and healthcare contexts, and locally appropriate training and assessment strategies should be developed where necessary [11,25].

Second, future research should generate stronger evidence on whether communication skills learned during training are sustained over time and effectively applied in real clinical practice. This requires longitudinal studies that follow learners across different stages of medical education and into professional practice, using outcome measures that extend beyond short-term OSCE performance [12,13,39,40,47].

Third, research needs to place greater emphasis on communication within healthcare teams and systems. As modern healthcare delivery is increasingly complex and multidisciplinary, effective team communication is essential for patient safety and quality of care, yet remains underexplored compared with clinician-patient interactions [4,6,48,49].

Finally, as telemedicine and artificial intelligence become routine components of healthcare, communication research should move beyond descriptive or exploratory work toward the rigorous evaluation of training approaches, ethical governance, and patient experiences in digital-first care environments [43,45].

## Conclusions

Over the past quarter-century, research on communication skills has evolved into a well-established, interdisciplinary, and globally distributed field. The literature demonstrates consolidation around established educational frameworks, increasing emphasis on assessment and outcomes, and growing responsiveness to digital and technology-mediated communication contexts. At the same time, persistent geographic and contextual gaps highlight the need for more inclusive, culturally grounded research to ensure that communication training models remain valid and effective across diverse healthcare systems.
